# Contained Rupture of the Ascending Aorta Caused by Primary Aldosteronism

**DOI:** 10.1016/j.atssr.2026.02.032

**Published:** 2026-03-18

**Authors:** Étienne Fasolt Richard Corvin Meinert, Arlene Körner, Willem Hendrik te Gussinklo, Alexander Brobeil, Matthias Karck, Bashar Dib

**Affiliations:** 1 Department of Cardiac Surgery, Heidelberg University Hospital, Heidelberg, Germany; 2 Department of Pathology, Heidelberg University Hospital, Heidelberg, Germany

## Abstract

A 36-year-old female patient presented with syncope and retrosternal chest pain. Computed tomography revealed contained rupture of the ascending aorta with no false lumen but an intraluminal thrombus. She underwent emergent open aortic repair. Postoperatively, refractory hypertension was noticeable. Upon further examination of the computed tomographic scan, a large right-sided adrenal mass became evident. Endocrinologic workup ruled out pheochromocytoma and revealed primary aldosteronism. She underwent robotic adrenalectomy, subsequently showed close to normal blood pressure values, and was discharged without further complications. We present here a rare case of contained aortic rupture in the setting of primary aldosteronism.

Primary aldosteronism is common and an underrecognized cause of arterial hypertension.^1^ It may predispose to severely increased cardiovascular risk,^2^ including aortic aneurysm and dissection.^3^ We present a case of contained aortic rupture—with neither sign of preexisting aortic aneurysm nor dissection—as the first manifestation of primary aldosteronism caused by right-sided adrenal adenoma in a young woman.

A 36-year-old woman presented to the emergency department after a syncopal episode accompanied by severe retrosternal chest pain radiating to the neck. She had experienced similar episodes over the preceding days. Her medical history was unremarkable apart from arterial hypertension. She had no family history of connective tissue disorders, and no phenotypical features were evident in the patient. Electrocardiogram and cardiac troponin values showed no signs of myocardial ischemia. A computed tomographic angiogram revealed a contained rupture of the ascending aorta, an adjacent intraluminal thrombus in the ascending aorta (Figure 1A), pericardial effusion, severely hypertrophic left ventricular myocardium, and incidentally a right adrenal mass measuring 64 × 53 mm (Figure 1B).

**Figure 1:**
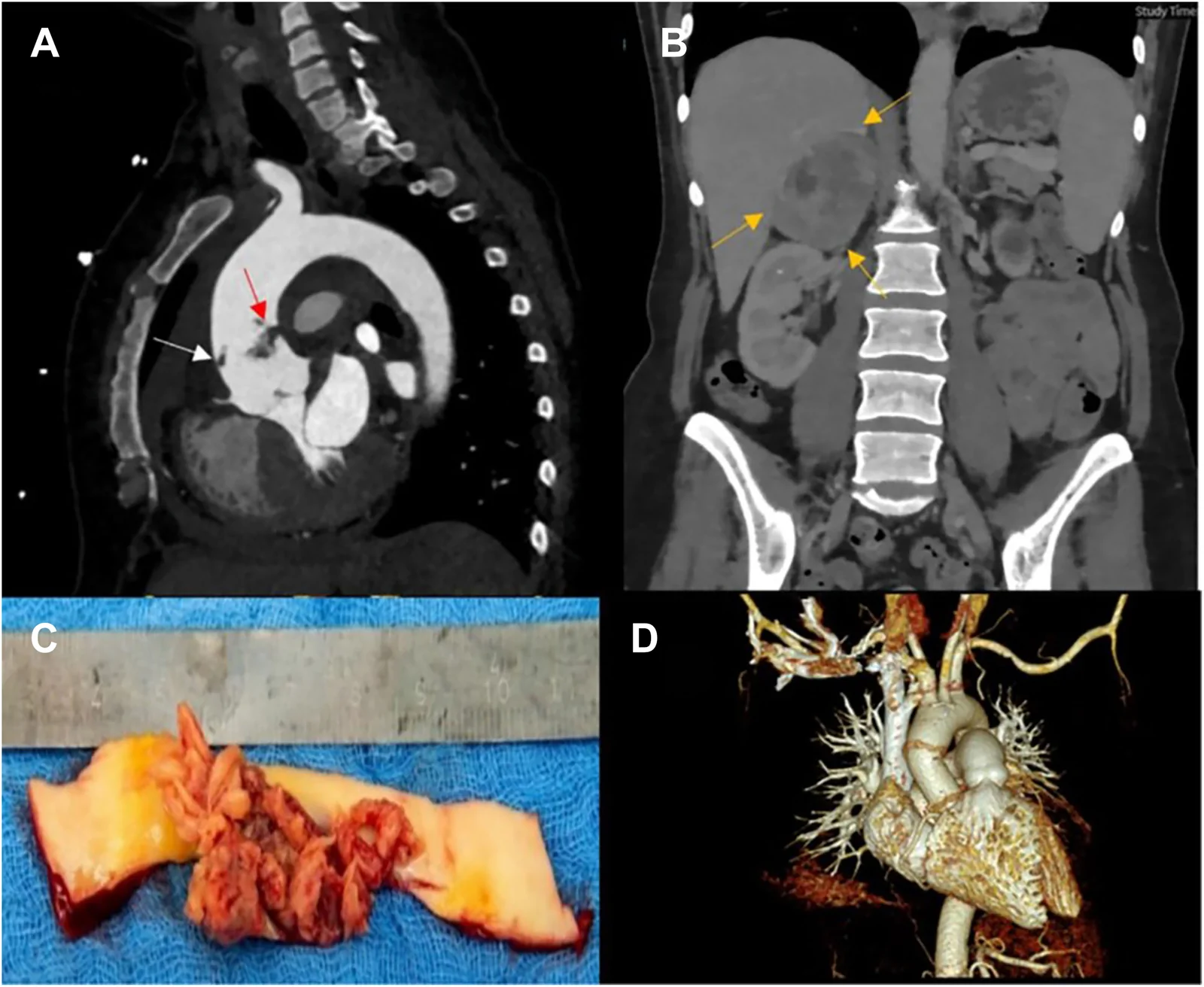
(A) Computed tomography shows aortic wall injury (white arrow) and an intramural thrombus (red arrow). (B) Incidental right adrenal mass (arrows). (C) Gross pathology of a part of the injured wall of the ascending aorta, including a mural thrombus. (D) Three-dimensional computed tomography reconstruction of the thoracic aorta shows the replacement of the ascending aorta.

She was transferred to the cardiac surgery department to undergo emergent aortic repair. Intraoperative transesophageal echocardiography confirmed the presence of a floating pedunculated mural thrombus in the ascending aorta and revealed concentric left ventricular hypertrophy, with an interventricular septal thickness of 24 mm. After median sternotomy, the pericardium was opened, and ∼600 mL of hemorrhagic pericardial effusion was evacuated.

The proximal aortic arch was cannulated. A 2-stage cannula was inserted into the right atrium for venous return. Cooling to a target temperature of 34 °C was commenced. After aortic cross-clamping and antegrade administration of Bretschneider cardioplegic solution, the ascending aorta was opened. A transversal intimal tear, measuring ∼4 cm, was identified in the proximal ascending aorta. Although no mobile intimal flap or false lumen was observed, there was a subadventitial hematoma at the outer portion of the tear site. A thrombus was present along the base of the tear inside the aortic lumen (Figure 1C). The ascending aorta was resected, and supracoronary replacement of the ascending aorta was performed using a 24-mm Dacron (Getinge) graft (Figure 1D).

The patient was weaned off bypass and sternal closure was performed in standard fashion. The total procedure time was 143 minutes. She was transferred to the intensive care unit postoperatively, was in a hemodynamically stable condition, and had no neurologic deficits. Despite treatment with 5 antihypertensive agents, hypertension persisted postoperatively. Considering the right adrenal mass detected in the preoperative computed tomographic scan, further endocrinologic workup was performed.

Plasma free metanephrine levels were within normal range, whereas 24-hour urinary fractionated metanephrine levels were slightly elevated. The result of a clonidine suppression test was negative. Hence, pheochromocytoma was ruled out. The refractory hypertension and hypokalemia, despite potassium administration, raised the suspicion for primary aldosteronism. The aldosterone-to-renin ratio was measured despite intake of several antihypertensive drugs, such as β-blockers, diuretics, and angiotensin receptor blockers. Aldosterone was 19.7 ng/dL, and the direct renin concentration was <0.5 mU/L and thus maximally suppressed. Aldosterone suppression testing was therefore omitted in this setting. Adrenal venous sampling was not performed, because the patient was young (36 years old) and a large (6.4 cm × 5.3 cm) right-sided adrenal adenoma (>1.0 cm) was evident on computed tomography.^4^

Primary aldosteronism caused by a right sided adrenocortical adenoma was the most likely diagnosis. The patient underwent robotic-assisted, laparoscopic right adrenalectomy. Histopathologic analysis confirmed an adrenocortical adenoma (Figure 2). The patient recovered normally and was discharged in stable normotensive condition on low-dose oral medication with bisoprolol. At the 1-year follow-up, the patient was doing well with arterial blood pressure values in a normal range.

**Figure 2:**
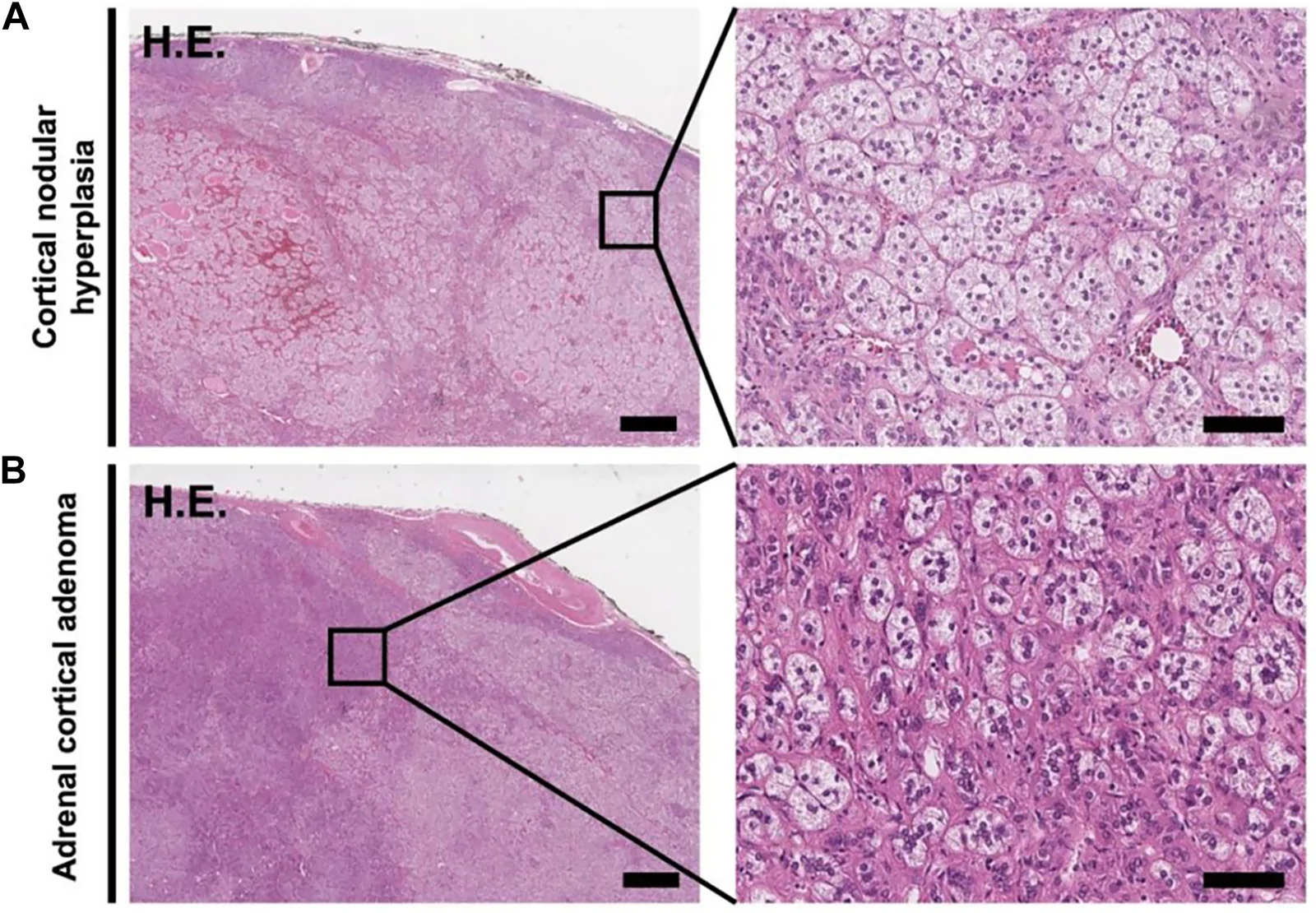
Histopathologic images of tissue of the right adrenal gland show (A) cortical nodular hyperplasia indicative of (B) adrenal cortical adenoma. Scale bar on the left = 500 μm, scale bar on the right = 150 μm. (H.E., hematoxylin and eosin.)

## Comment

To sum up, we present a case of a 36-year-old female patient with a history of hypertension and presenting with contained rupture of the ascending aorta with an adjacent intraluminal thrombus. She underwent emergent open aortic repair. Postoperative workup of refractory hypertension yielded the diagnosis of primary aldosteronism caused by a right-sided adrenal adenoma. Upon adrenalectomy, the patient recovered without complications, and normalization of blood pressure values was noted.

Several authors have reported on aortic dissection caused by primary aldosteronism.^5^^,^^6^ Based on this knowledge, there have even been studies linking plasma aldosterone concentration to aortic dissection and aneurysm^7^ and genetically predicted primary aldosteronism to aortic aneurysm or aortic dissection.^3^ Moreover, recent meta-analyses have shown that patients with primary aldosteronism also bear a higher risk of diabetes, stroke, coronary artery disease, atrial fibrillation, and heart failure compared with patients with essential hypertension.^2^

Primary aldosteronism is still widely underrecognized (5%-10% of all patients with hypertension^1^), subjecting potentially treatable patients to these mentioned risks. Morbidity and mortality can largely be mitigated by effective treatment of primary aldosteronism by adrenalectomy or mineralocorticoid receptor antagonists. Recent guidelines have even recommended screening all patients with diagnosed hypertension for primary aldosteronism.^4^^,^^8^

Our case is a strong argument in line with this recommendation. Aortic rupture was the first complication of hypertension caused by primary aldosteronism in this young patient. Neither an aneurysm nor dissection preceded it. She only survived because the rupture was contained by the adventitia and visceral pericardium. Failure of this final layer would almost certainly have resulted in a fatal outcome. Based on this experience of contained aortic rupture—without aneurysm or dissection—caused by primary aldosteronism, we strongly endorse more widespread screening efforts for primary aldosteronism, especially in young individuals.
